# Efficacy of a Smartphone App in Enhancing Medication Adherence and Accuracy in Individuals With Schizophrenia During the COVID-19 Pandemic: Randomized Controlled Trial

**DOI:** 10.2196/50806

**Published:** 2023-12-14

**Authors:** Huan Hwa Chen, Hsin Tien Hsu, Pei Chao Lin, Chin-Yin Chen, Hsiu Fen Hsieh, Chih Hung Ko

**Affiliations:** 1 School of Nursing, Chung Hwa University of Medical Technology Tainan Taiwan; 2 College of Nursing, Kaohsiung Medical University Kaohsiung Taiwan; 3 Department of Medical Research, Kaohsiung Medical University Hospital, Kaohsiung Medical University Kaohsiung Taiwan; 4 Department of Biomechatronics Engineering, National Pingtung University of Science and Technology Pingtung Taiwan; 5 Center for Long-Term Care Research, Kaohsiung Medical University Kaohsiung Taiwan; 6 Kaohsiung Chang Gung Memorial Hospital Kaohsiung Taiwan

**Keywords:** cognitive functions, medication adherence, psychiatric symptoms, schizophrenia, smartphone app

## Abstract

**Background:**

Poor medication adherence or inaccuracy in taking prescribed medications plays an important role in the recurrence or worsening of psychiatric symptoms in patients with schizophrenia, and the COVID-19 pandemic impacted their medication adherence with exacerbated symptoms or relapse. The use of mobile health services increased during the COVID-19 pandemic, and their role in improving mental health is becoming clearer.

**Objective:**

This study aimed to explore the effectiveness of a smartphone app (MedAdhere) on medication adherence and accuracy among patients with schizophrenia and to measure their psychiatric symptoms and cognitive functions.

**Methods:**

In this 12-week experimental study, participants were provided interventions with the MedAdhere app, and data were collected between June 2021 and September 2022. A total of 105 participants were randomly assigned to either the experimental or control groups. We used the Positive and Negative Syndrome Scale and Mini-Mental State Examination to measure the participants’ psychiatric symptoms and cognitive functions. Generalized estimating equations were used for data analysis.

**Results:**

A total of 94 participants met the inclusion criteria and completed the protocol, and the medication adherence rate of the experimental group was 94.72% (2785/2940) during the intervention. Psychotic symptoms (positive, negative, and general psychopathology symptoms) and cognitive functions (memory, language, and executive function) were significantly improved in the experimental group compared to the control group after the intervention.

**Conclusions:**

The MedAdhere app effectively and significantly improved medication adherence and, thereby, the psychiatric symptoms of patients with schizophrenia. This artificial intelligence assisted app could be extended to all patients who need to be reminded to take medication on schedule.

**Trial Registration:**

ClinicalTrials.gov NCT05892120; https://clinicaltrials.gov/study/NCT05892120

## Introduction

### Overview

Schizophrenia is a mental disorder that imposes a heavy economic burden and causes severe functional disabilities, including cognitive impairment [1,2]. Its clinical manifestations are divided into positive and negative symptoms. Positive symptoms include delusions, hallucinations, and disorganized behavior, which are often accompanied by impaired cognitive and socio-occupational functions, while in contrast, negative symptoms manifest as apathy and decreased motivation, which gradually become apparent as the disorder progresses [3]. Many factors accelerate aging in patients with schizophrenia, making their life span shorter than that of individuals without schizophrenia, with impacts such as deterioration of life functioning, the presence of chronic illnesses, obesity, and an unhealthy lifestyle [4]. Male and female patients with schizophrenia have an average life expectancy of 61.48 years and 60.81 years, respectively [5].

Schizophrenia is a treatable disorder, and early intervention can prevent further deterioration. Antipsychotic drugs are the first choice for alleviating the psychiatric symptoms of these patients, and the goal of regular medication with the right drugs, dosages, and frequency is to prevent recurrence and overall functional deterioration [6,7]. Although it is common for patients with schizophrenia to not adhere to their prescriptions for a variety of reasons, including the stigma of mental illness, side effects of drugs, forgetting to take medicine, and lack of insight [8,9].

The medication adherence rate among patients diagnosed with schizophrenia for the first time is only 50% in the first year [4,5], and the rate of relapse within 5 years is up to 80% [10]. Similar studies have reported that 50% to 80% of patients with schizophrenia do not take their medications regularly [6,7] or take the wrong medications or dosages [8]. Nonadherence may lead to the deterioration of symptoms, a decline in daily life functions, and a poor prognosis; it may also result in hospitalization and the escalation of medical expenses [9,11].

The COVID-19 pandemic has affected social contact patterns in various situations, including in the field of medical services. Individuals with mental disorders exhibited higher levels of concern regarding COVID-19 and fear of getting infected [12], with the result that 64% of them had worsening of their symptoms, 39% were unable to receive treatment, and 38% missed their medications [13]. Subsequently, wider use of telehealth and digital tools was promoted, such as smartphone apps, in providing medical services for the reduction of person-to-person contact to lower the likelihood of COVID-19 transmission to patients [14]. Poor medication adherence was one of the major causes of the increasing relapse rate among patients with schizophrenia during the COVID-19 pandemic [15], and it is crucial to use remote monitoring to track oral medication adherence to lower relapse rates or the deterioration of psychiatric symptoms.

An increasing number of smartphone apps are being developed to care for and train patients with schizophrenia [16,17], which include apps for improving patients’ psychiatric symptoms, medication adherence, and social and cognitive functions [17-19]. Most of the apps for medication adherence in patients with schizophrenia are designed to remind patients to take their medications on time [19,20]. A smartphone app named “MedAdhere” was developed by Chen et al [21] to observe the medication adherence of these patients with recognition of the appearance of the prescribed antipsychotic drugs as well as drug-taking behavior through the smartphone camera [21].

### Study Aims

This study aimed to explore the effectiveness of intervention with the MedAdhere app on medication adherence and accuracy in patients with schizophrenia and to measure their psychiatric symptoms and cognitive functions during the period of the COVID-19 pandemic. We hypothesized that using the MedAdhere app would improve medication adherence, psychiatric symptoms, and cognitive function in patients with schizophrenia compared to the control group.

## Methods

### Study Design

The protocol was registered with ClinicalTrials.gov (NCT05892120) at the time of submission. We used an experimental design, using random sampling with each daycare center as a unit, and assigned patients in these centers to either the experimental or control group using a random number generator. This study is single-blinded, with research assistants being the blinded party. All participants received the usual care, while participants in the experimental group additionally downloaded and used the MedAdhere app on their personal smartphones during the 12-week study.

### Participants and Setting

The sample size was calculated using G*Power (version 3.1.1; Axel Buchner) for the *F*_1_ test and its repeated measures with an effect size of 0.25, a significance level of .05, and a power (1 – β) of 0.80; the estimated minimum sample size was 68 participants [22], with all participants being recruited from a psychiatric daycare center across 2 medical centers and 1 regional hospital.

There are various treatment modalities for patients with schizophrenia based on each patient’s psychiatric condition, including acute psychiatric units, chronic wards, daycare centers, and community services, and they may be transferred from one treatment modality to another based on their condition. Patients in daycare were transferred from acute wards where they received initial treatment, and after a period of time, they were treated well with a relatively stabilized mental status under a suitable medication and dosage. The care in the daycare center mainly focuses on regular medication and psychosocial rehabilitation. Daycare for psychiatric patients can be effective in returning the patient to a normal life in the community or relieving the strain on a family [23]. The daycare center is an important psychiatric rehabilitation unit for patients with stable psychiatric symptoms who are suitable users of this app.

A total of 105 patients participated in this study, and they were randomly assigned to the experimental or control group, although 11 patients in the experimental group did not complete this study; finally, 94 participants completed all processes of the study protocol (Multimedia Appendix 1).

The inclusion criteria were as follows: (1) patients diagnosed with schizophrenia by a psychiatrist according to the *Diagnostic and Statistical Manual of Mental Disorders, Fifth Edition* [3]; (2) aged between 20 and 65 years; (3) able to read traditional Chinese; (4) owned a smartphone; and (5) currently admitted to a psychiatric daycare center. The exclusion criteria were as follows: patients with intellectual disability or severe cognitive function impairment (Mini-Mental State Examination [MMSE] score <17) [24].

### Study Protocol

#### Control Group

Medications for the control group are divided into 2 parts: medication in the daytime and nighttime. Daytime medication at the daycare center is assisted and supported by daily activities arranged by the staff. Nighttime medication is taken by the patient without any intervention.

#### Experimental Group

Medications for the experimental group are divided into 2 parts: medication in the daytime and nighttime. Daytime medication at the daycare center is assisted and supported by daily activities arranged by the staff. Intervention for nighttime medication is conducted with the MedAdhere app.

**Figure 1:**
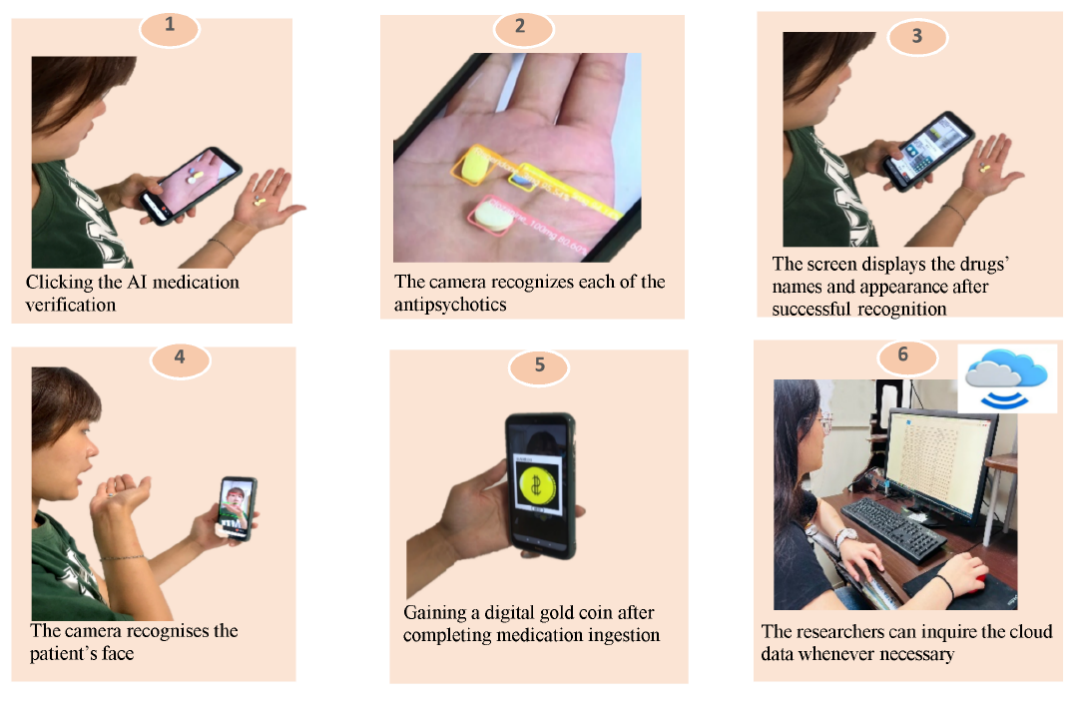
Steps of antipsychotic ingestion recognised by MedAdhere app.

**Figure 2:**
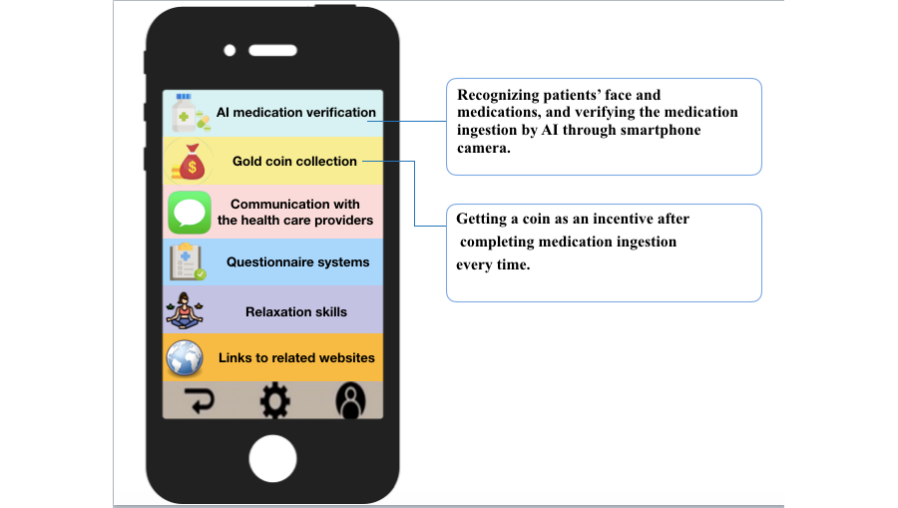
Home page of the MedAdhere app. AI: artificial intelligence.

Before the intervention, researchers first explained the functions of the MedAdhere app to the patient and set the patient’s antipsychotic medication details on their smartphones, including type, dosage, and frequency, and then completed the recognition of the patient’s face and the appearance of all antipsychotics. Then, each patient was taught how to use the app step by step (Figure 1) by providing operation manual practical training. The intervention stage began, and we confirmed that the patient knew how to operate the app. In addition to the verification of medication status using the MedAdhere app, we used a bidirectional interaction approach where patients could ask researchers questions on the web through the Line app (NHN Japan; which is one of the related links in the MedAdhere app), and the team would send messages to or call the patient when any abnormalities were detected. The home page of the MedAdhere app is shown in Figure 2.

Figure 1 shows the steps of antipsychotic ingestion by patients recognized by the MedAdhere app, and any errors can be detected using this app. For example, if the participant took the medication at the wrong time or took the wrong drug or dosage, this MedAdhere app promptly detected such errors and uploaded them to the cloud, while the results of antipsychotic ingestion were also recorded by this app and uploaded to a cloud server. The MedAdhere app could also be used offline and automatically upload all records to the cloud server when connected to the internet. Researchers and assistants had the ability to access cloud data whenever necessary to observe the patient’s medication status, in addition to 2 routine check-in times at 10 PM and 11 PM.

### Data Collection

The data were collected between June 2021 and September 2022. Demographic characteristics, including age, gender, marital status, years of education, living status, and religious beliefs, were gathered. The symptoms and cognitive functions of all participants were measured using the Positive and Negative Syndrome Scale (PANSS) and MMSE at baseline and at the end of the intervention, respectively.

### Instruments

#### MedAdhere App

The MedAdhere app is a medication management app available on Android platforms for patients’ medication scheduling, reminders, tracking, medication adherence assessments and recognition of the patient’s face and the appearance of antipsychotics. When the participant takes the medication at the wrong time or takes the wrong drug or dosage, this MedAdhere app can detect these errors, upload such errors to the cloud, and trigger an alarm for the participant [21].

#### Medication Adherence Rate

Medication adherence is defined as the proportion of the administered drug doses in relation to the prescribed doses taken by the patient within a specific time frame. Based on this definition, we calculated the patient’s medication adherence rate of the control group by counting their pills left at the end of intervention, and the patient’s medication adherence rate of the experimental group was calculated from the data in the cloud. In addition to antipsychotics, there was no antidepressant prescribed for any of our participants, but there were other psychotropic medications prescribed for our participants, and these adjunctive drugs were not included to be identified by our MedAdhere app.

#### PANSS Instrument

The PANSS is among the best-validated instruments for assessing positive, negative, and general psychopathological symptoms associated with patients with schizophrenia. It is a standardized clinical interview that rates the presence and severity of positive and negative symptoms as well as general psychopathological symptoms (such as anxiety and depression) within the past week for patients with schizophrenia. Of the 30 items on the scale, 7 are positive symptoms, 7 are negative symptoms, and 16 are general psychopathological symptoms. The symptom severity for each item is rated according to the anchoring points on a 7-point scale (1=absent and 7=extreme) that best describes the symptom [25]. The PANSS score is the sum of ratings across items, ranging from 7 to 49 for the PANSS and 16 to 112 for the general psychopathology scale. Higher scores indicate more severe symptoms [26]. The Cronbach α was .86 [27].

#### MMSE Instrument

Cognitive tests were conducted using the MMSE [24]. The MMSE is, in some way, the best-known and most widely used measure of cognition in clinical practice worldwide. The MMSE consists of 15 questions that assess the following 7 cognitive domains: orientation to time (5 points), place (5 points), memory registration and recall (3 points), attention and calculation (8 points), language (5 points), reasoning and problem-solving (3 points), and executive function (1 point). The total MMSE score ranges from 0 to 30, with higher scores indicating better cognitive function. The internal consistency of the MMSE method was represented by Cronbach α at the level of .78 [28].

### Data Analysis

Descriptive analyses by chi-square and Mann-Whitney *U* test were performed using SPSS software (version 21.0; SPSS Inc). Descriptive analysis was based on participants’ demographic characteristics, such as age, gender, education, marital status, and religious beliefs. After controlling the confounding factor of age, the generalized estimating equation was used to assess psychotic symptoms (positive, negative, and general psychopathological symptoms) and cognitive functions (orientation, memory, attention and calculation, language, reasoning and problem-solving, and executive function) from baseline to the end of intervention in the 2 groups. All tests were 2-tailed, and *P*<.05 was considered significant.

### Ethical Considerations

This study was approved by the institutional review board of the Kaohsiung Medical University Hospital: KMUHIRB-SV (I)–20200096. The principal investigator visited each psychiatric daycare center, explained the study’s purpose and process to all participants, and inquired regarding their willingness to participate. Written informed consent was obtained from all participants, and they were informed that they could withdraw from the study at any time without providing a reason. Each participant was offered NT $400 (US $13-14) as compensation for their participation.

## Results

### Baseline Data

All participants had access to their private mobile networks (4G LTE). A total of 105 participants were randomly assigned to the experimental or control group, and 94 completed all the processes of our protocol. The demographic characteristics of the respondents are presented in Table 1.

**Table 1:** Demographic characteristics and baseline outcomes.

Characteristics			Control group (n=59)		Experimental group (n=35)		Chi-square test or Mann-Whitney *U* Test		*P* value
Age (years), mean (SD)			50.45 (11.4)		41.29 (10.5)		–1.587^a^		.11
**Gender, n (%)**							0.980^b^		.32
	Male	28 (47.5)		20 (57.1)					
	Female	31 (52.5)		15 (42.9)					
**Education, n (%)**							0.223^b^		.90
	Junior high school	6 (10.2)		4 (11.5)					
	Senior high school	37 (62.7)		20 (57.1)					
	≥College	16 (27.1)		11 (31.4)					
**Marital status, n (%)**							0.826^b^		.66
	Single	47 (79.7)		28 (80.0)					
	Married	8 (13.6)		6 (17.1)					
	Divorced	4 (6.7)		1 (2.9)					
**Living status, n (%)**							2.669^b^		.45
	Alone	11 (18.6)		5 (14.3)					
	Spouse	2 (3.14)		1 (2.8)					
	Friends	2 (3.14)		0 (0)					
	Parent or child	44 (74.6)		29 (82.9)					
**Religious belief, n (%)**							0.159^b^		.69
	No	17 (28.8)		10 (28.6)					
	Yes	42 (71.2)		25 (71.4)					
**PANSS^c^, mean (SD)**									
	Positive symptoms	46.36 (4.1)		49.41 (3.9)		–0.526^a^		.60	
	Negative symptoms	46.38 (3.6)		49.39 (3.7)		–0.519^a^		.60	
	General psychopathology symptoms	44.10 (6.2)		53.23 (7.0)		–1.572^a^		.11	
**MMSE^d^, mean (SD)**									
	Orientation	46.08 (1.7)		49.89 (0.4)		–0.974^a^		.33	
	Attention and calculation	48.00 (1.7)		46.66 (1.6)		–0.247^a^		.81	
	Language	48.87 (0.6)		45.19 (0.8)		–0.889^a^		.37	
	Reasoning and problem-solving	47.53 (0.7)		47.46 (0.4)		–0.018^a^		.99	
	Memory	46.92 (0.7)		48.47 (0.7)		–0.329^a^		.74	
	Executive function	46.83 (0.4)		48.63 (0.3)		–0.517^a^		.61	

^a^Chi-square test (*df*).

^b^Mann-Whitney *U* test.

^c^PANSS: Positive and Negative Syndrome Scale.

^d^MMSE: Mini-Mental State Examination.

### Medication Adherence Rate

The medication adherence rates were 94.72% (2785/2940; total doses taken by all patients over 12 weeks / [35 person ×12 weeks × 7 days × doses] × 100%) and 64.43% (3193/4956; total doses taken by all patients over 12 weeks / [59 person ×12 weeks × 7 days × doses] × 100%) in the experimental group and the control group, respectively. The medication adherence rate of the experimental group was higher than the control group during the intervention (Table 2).

**Table 2:** Medication adherence rate.

Group	At 8 weeks, n/N (%)	At 12 weeks, n/N (%)
Control group (n=59)	2111/3304 (63.89)	3193/4956 (64.43)
Experimental group (n=35)	1764/1960 (90)	2785/2940 (94.72)

### Changes in Psychiatric Symptoms and Cognitive Functions

The changes in psychiatric symptoms and cognitive functions in the 2 groups from baseline to the end of the intervention in 12 weeks are shown in Tables 3 and 4.

**Table 3:** Changes of psychiatric symptoms from baseline to the end of intervention.

Parameters			T2 vs T0		T1 vs T0	Age		EG^a^ × T2 vs CG^b^ × T2		EG × T1 vs CG × T1		EG × age
**Positive symptoms**												
	β	–2.33		.92		–.02	–.84		–1.95		.15	
	Mean	10.54		13.79		—^c^	12.03		10.92		—	
	SE	1.7		1.49		—	0.26		0.23		—	
	*P* value	.17		.54		—	.33		.02^d^		.74	
**Negative symptoms**												
	β	–2.59		–.43		–.01	–.92		–1.99		0	
	Mean	8.63		10.79		—	10.3		9.23		—	
	SE	1.5		1.64		—	0.38		0.36		—	
	*P* value	.09		.79		—	.24		.007^e^		.71	
**General psychopathology symptoms**												
	β	–4.63		1.98		–3.16	–4.37		–.03		–4.63	
	Mean	20.9		27.51		—	21.93		21.16		—	
	SE	2.97		2.62		—	0.18		0.15		—	
	*P* value	.12		.45		—	.02^d^		<.001^f^		.65	

^a^EG: experimental group.

^b^CG: control group.

^c^Not available.

^d^*P*<.05.

^e^*P*<.01.

^f^*P*<.001.

**Table 4:** Changes of cognitive functions from baseline to the end of the intervention.

Parameter		T2 vs T0	T1 vs T0	Age	EG^a^ × T2 vs CG^b^ × T2	EG × T1 vs CG × T1	EG × age
**Orientation**							
	β	–.10	–.32	–2.5E	.25	.25	.00
	Mean	9.68	9.46	—^c^	10.03	10.03	—
	SE	0.63	0.63	0.01	0.26	0.26	0.01
	*P* value	.87	.53	—	.35	.28	.65
**Attention and calculation**							
	β	.72	–.04	.00	.19	.16	–.00
	Mean	7.46	6.70	—	6.93	6.90	—
	SE	0.73	0.73	0.02	0.02	0.02	0.02
	*P* value	.32	.95	—	.62	.66	.61
**Memory**							
	β	.30	–.26	.01	–.07	.31	–.02
	Mean	2.64	2.08	—	2.27	2.65	—
	SE	0.37	0.37	0.01	0.01	0.01	0.01
	*P* value	.42	.28	—	.71	.03^d^	.02^d^
**Language**							
	β	.19	.18	.01	.41	.28	.00
	Mean	4.74	4.73	—	4.96	4.83	—
	SE	0.34	0.34	0.01	0.01	0.01	0.01
	*P* value	.57	.64	—	.16	.16	.86
**Reasoning and problem-solving**							
	β	.14	–.28	.00	.18	–.01	–.01
	Mean	2.79	2.37	—	2.83	2.65	—
	SE	0.25	0.25	0.00	0.00	0.00	0.00
	*P* value	.58	.30	—	.16	.97	.03^d^
**Executive function**							
	β	–.45	–.18	–.00	.33	.18	.00
	Mean	0.59	0.84	—	1.35	1.20	—
	SE	0.22	0.22	0.00	0.00	0.00	0.00
	*P* value	.04^d^	.40	—	<.001^e^	.08	.80

^a^EG: experimental group.

^b^CG: control group.

^c^Not available.

^d^*P*<.05.

^e^*P*<.001.

After controlling the confounding factor of age, we found significant improvements in positive, negative, and general psychopathology symptoms in the experimental group, with a decrease of 0.143 in the positive (*P*=.02), 0.137 in the negative (*P*=.007), and 0.013 in the general psychopathological symptoms (*P*<.001) following an 8-week intervention with the MedAdhere app (Table 3). Additionally, there were significant improvements in the cognitive domains of memory, language, and executive function in the experimental group, with an increase of 1.51 in language (*P*=.012) and 1.39 in executive function (*P*<.001) after intervention with the MedAdhere app for 12 weeks (T2; Table 4).

## Discussion

### Overview

The policy of the government in Taiwan during the COVID-19 period was to reduce interpersonal contact against the transmission of coronavirus. In addition to the medication adherence of patients, this app was designed to reduce interpersonal contacts during the COVID-19 pandemic and to reduce the burden on health care providers.

### Main Findings

In this study, the MedAdhere app contributed to the medication adherence rate during the intervention, and this study also found that patients’ positive and negative symptoms and general psychopathology symptoms significantly improved after the intervention, even though the experimental group had more severe general psychopathology symptoms than the control group at baseline. In addition, there were significant differences in cognitive functions between the 2 groups.

### Medication Adherence

The estimated medication adherence rate was 50% among patients with schizophrenia who did not receive any intervention [29]. Another study reported an adherence rate of 65% with different interventions for the same purpose of enhancing medication adherence in patients [30]. A higher medication adherence rate essentially contributes to stabilizing the psychiatric symptoms of patients and lowering their readmission rates [31]. The medication adherence rate in the experimental group was 94.72% during the intervention using the MedAdhere app, higher than the control group’s 64.43% in this study. Our results suggest that the utility of the MedAdhere app for artificial intelligence verification of medication ingestion behavior plays an important role in enhancing patients’ medication adherence, and this is an easily implemented intervention [32].

### Psychiatric Symptoms

Our results showed that intervention with the MedAdhere app could significantly improve patients’ positive and negative symptoms in 12 weeks compared to the control group. This result was similar to that of a previous study showing that using the medication adherence app could improve positive and negative symptoms in patients with schizophrenia [33].

Negative symptoms should be treated by other rehabilitation modalities to improve pleasure, motivation, and cognitive skills relevant to apathy and anhedonia [34,35]. In addition to verifying medication ingestion behavior using the MedAdhere app, patients were able to ask questions and interact with researchers on the web during the intervention through the Line app, which is one of the related links on the MedAdhere app’s home page. Interactions between researchers and patients can be regarded as a form of social skill training that has been documented to have medium effects on reducing negative symptoms in individuals with schizophrenia [36], and such interactions might be one of the reasons for alleviating our participants’ negative symptoms of social withdrawal, blunted affect, emotional withdrawal, and a lack of spontaneity and flow of conversation.

### Cognitive Functions

Cognitive functions declined in the control group, whereas the cognitive functions of the experimental group improved significantly in the domains of memory, language, and executive function after the 12-week intervention. This could be attributed to the use of the MedAdhere app with novel technology, which provides repeated multisensory stimuli and rewards after each medication ingestion, which in turn enhances their motivation to continue using the app.

Cognitive function in patients with schizophrenia can be improved through exercise [37], quality sleep [38], and symptomatic stability [39]. Our results may have been affected by the patient’s sleep patterns [38] and symptomatic stability [39], both of which are considered to be improved by antipsychotics. Compared to conventional methods for medication adherence that require routine monitoring by family members, telephone visits by home health care providers, or monthly visits by clinicians, this innovative MedAdhere app effectively empowers patients’ medication adherence and reduces the burden on health care providers. Such an artificial intelligence-assisted adherence-enhancing app can be extended to all patients who require regular medication but may forget to take their medication on time, for the benefits of better adherence and to prevent the intake of the wrong medication or duplicating doses.

The MedAdhere app requires minimal training for implementation. To ensure that patients can continue using this app, we initially designed the app interface to be user-friendly and easy to operate, with the added incentive of earning a digital gold coin upon completing medication ingestion as a reward. In addition, we have established alliances with clinical professionals and family members, providing the app and cloud access to health care teams and allowing them to keep monitoring patients’ medication adherence. With the support of patients’ families, each participant can keep using this MedAdhere app after the intervention.

### Limitations

This study had some limitations. First, there might be selection bias because the MedAdhere app can only be installed on the Android platform. Patients who did not own a smartphone or whose smartphones were not Android system based were excluded from this study. Second, this was a single-blinded design with a 12-week study duration, and a double-blinded study design with long-term follow-up is indicated to reduce potential bias and confirm the effects of the MedAdhere app on improving psychiatric symptoms and cognitive functions in patients with schizophrenia. Third, we did not calculate how many times or the frequency of communication between participants and our team. In the future, it will be possible to calculate the number of bidirectional interactions. Fourth, the MedAdhere app has only been used in Taiwan thus far and can be translated into other languages for use in other countries in the future. Finally, there were other psychotropic medications prescribed for our participants, and these adjunctive drugs were not included nor identified by our MedAdhere app.

### Conclusions

The novelty of this study is the development of the MedAdhere app using a smartphone’s camera to recognize patients’ faces, drugs, and medication-taking behavior. This accessible tool has the potential to significantly improve medication adherence in patients with schizophrenia. The additional value of this MedAdhere app is to assist associated health care providers or partners’ families in confirming patients’ medication status to reduce the risk of relapse or repeated hospitalization.

## Appendix

Multimedia Appendix 1 Study protocol.

Multimedia Appendix 2 CONSORT-EHEALTH checklist.

## Abbreviations

MMSE: Mini-Mental State Examination
PANSS: Positive and Negative Syndrome Scale
