# Supplementary figures and images for: Efficacy of a Smartphone App in Enhancing Medication Adherence and Accuracy in Individuals With Schizophrenia During the COVID-19 Pandemic: Randomized Controlled Trial
Source: JMIR Ment Health. 2023 Dec 14;10:e50806. doi: 10.2196/50806 (PMC10727482; doi:10.2196/50806)

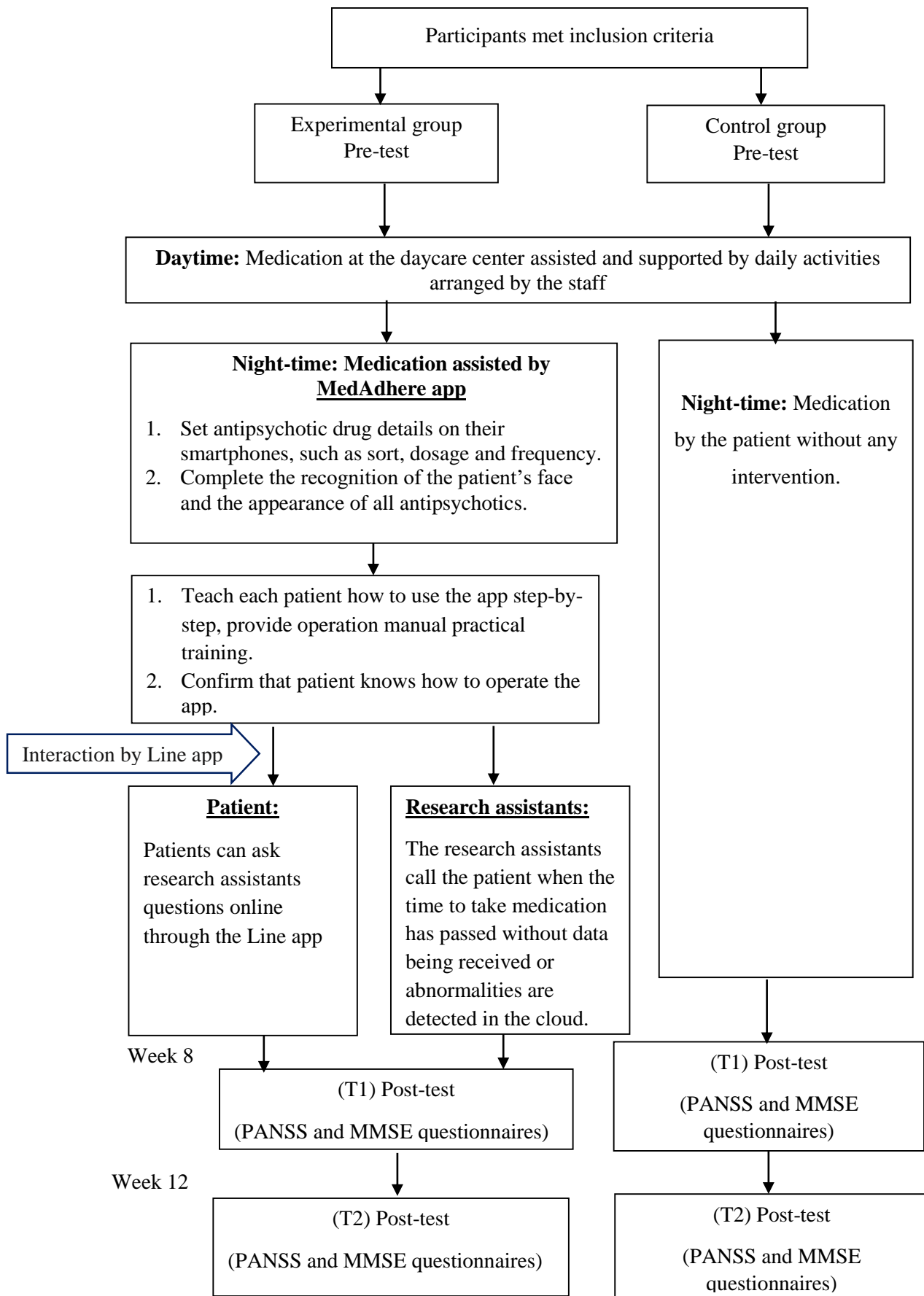

**Figure 1.** Study protocol

Supplement: Multimedia Appendix 1 [file mental_v10i1e50806_app1.pdf]
